# Cellular infiltrates and injury evaluation in a rat model of warm pulmonary ischemia–reperfusion

**DOI:** 10.1186/cc2992

**Published:** 2004-11-10

**Authors:** Bart P Van Putte, Jozef Kesecioglu, Jeroen MH Hendriks, Veerle P Persy, Erik van Marck, Paul EY Van Schil, Marc E De Broe

**Affiliations:** 1 Department of Thoracic and Vascular Surgery, University Hospital Antwerp, Antwerp, Belgium; 2 Department of Cardiothoracic Surgery, University Medical Center, Utrecht, The Netherlands; 3 Intensive Care Center, University Medical Center, Utrecht, The Netherlands; 4 Division of Perioperative Medicine and Emergency Care, University Medical Center, Utrecht, The Netherlands; 5 Department of Pathology, University Hospital Antwerp, Antwerp, Belgium; 6 Department of Nephrology, University Hospital Antwerp, Antwerp, Belgium

**Keywords:** acute lung injury, acute respiratory distress syndrome, neutrophils, T cells, warm pulmonary ischemia–reperfusion injury

## Abstract

**Introduction:**

Beside lung transplantation, cardiopulmonary bypass, isolated lung perfusion and sleeve resection result in serious pulmonary ischemia–reperfusion injury, clinically known as acute respiratory distress syndrome. Very little is known about cells infiltrating the lung during ischemia–reperfusion. Therefore, a model of warm ischemia–reperfusion injury was applied to differentiate cellular infiltrates and to quantify tissue damage.

**Methods:**

Fifty rats were randomized into eight groups. Five groups underwent warm ischemia for 60 min followed by 30 min and 1–4 hours of warm reperfusion. An additional group was flushed with the use of isolated lung perfusion after 4 hours of reperfusion. One of two sham groups was also flushed. Neutrophils and oedema were investigated by using samples processed with hematoxylin/eosin stain at a magnification of ×500. Immunohistochemistry with antibody ED-1 (magnification ×250) and antibody 1F4 (magnification ×400) was applied to visualize macrophages and T cells. TdT-mediated dUTP nick end labelling was used for detecting apoptosis. Statistical significance was accepted at *P *< 0.05.

**Results:**

Neutrophils were increased after 30 min until 4 hours of reperfusion as well as after flushing. A doubling in number of macrophages and a fourfold increase in T cells were observed after 30 min until 1 and 2 hours of reperfusion, respectively. Apoptosis with significant oedema in the absence of necrosis was seen after 30 min to 4 hours of reperfusion.

**Conclusions:**

After warm ischemia–reperfusion a significant increase in infiltration of neutrophils, T cells and macrophages was observed. This study showed apoptosis with serious oedema in the absence of necrosis after all periods of reperfusion.

## Introduction

Ischemia–reperfusion injury in lung tissue is a common problem in medical practice, with sometimes severe consequences such as acute respiratory distress syndrome (ARDS) and a high mortality rate for the patient. Some causes of warm ischemia–reperfusion injury are cardiopulmonary bypass during cardiac surgery and pulmonary sleeve resection. In contrast, lung transplantation is the main example of partial cold ischemia–reperfusion injury.

Neutrophils are known to be one of the cell types responsible for tissue damage in many ways. First, they are able to deliver toxic radicals that damage pulmonary endothelium directly or indirectly by activating caspase-3, which results in apoptosis [1,2]. Second, they can damage pulmonary endothelium and parenchyma by delivering elastase and other proteases [3]. Third, the cell membrane of activated neutrophils becomes rigid and adhesion between neutrophils and endothelial adhesion molecules occurs, resulting in sequestration and a 'no-reflow phenomenon' [4,5].

The role of neutrophils in pulmonary ischemia–reperfusion injury has also been investigated in experiments in which neutrophil depletion was induced and by the inhibition of tissue infiltration. The role of the neutrophil is currently still controversial [6-9].

The role of macrophages has been investigated in several transplantation models [3,10,11]. Eppinger and colleagues have specified chemical mediators of reperfusion injury by using antibodies against cytokines. Although some mediators seemed to be required during the early phase of ischemia–reperfusion injury, only tumor necrosis factor-α (TNF-α) is involved in the evolution of late ischemia–reperfusion injury. These cytokines are released from activated macrophages probably as a result of acute lung reperfusion [10]. These results suggest a role for macrophages in the early reperfusion phase and a role for activated and recruited neutrophils in the late reperfusion phase [3]. Currently, the role of lymphocytes in ischemia–reperfusion injury remains unclear. Qayumi and colleagues concluded that upregulation of MHC II on peripheral lymphocytes is related to the degree of damage caused by ischemia–reperfusion [12].

Apoptosis, necrosis and alveolar oedema, representing alveolar permeability, are morphological changes of ischemia–reperfusion-induced lung injury. Fischer and colleagues were the first to describe apoptosis of specifically type II alveolar pneumocytes resulting from pulmonary ischemia–reperfusion in a human lung transplantation study [13].

In summary, little is known about the role of neutrophils, T cells and macrophages in ischemia–reperfusion injury. In preparation for studies investigating the specific role of infiltrating cells, the aim of this study was to specify the type of infiltrating cells and their sequence after 1 hour of warm ischemia followed by 30 min to 4 hours of reperfusion in a model of acute lung injury, which was defined by quantifying apoptosis and alveolar oedema.

## Materials and methods

### Animals

Male inbred Wistar rats (mean weight 225 g), obtained from Iffa Credo (Brussels, Belgium), were used for all experiments. Animals were treated in accordance with the Animal Welfare Act and the *Guide for the Care and Use of Laboratory Animals *(NIH Publication 86-23, revised 1985). The rats were transported in sterile conditions, housed in suspended mesh-wired cages and fed *ad libitum *with a standard pellet diet (standard rat chow; Hope Farms, Woerden, The Netherlands). The Ethical Committee of the University of Antwerp approved the experimental protocols.

### Study design

**Figure 1:**
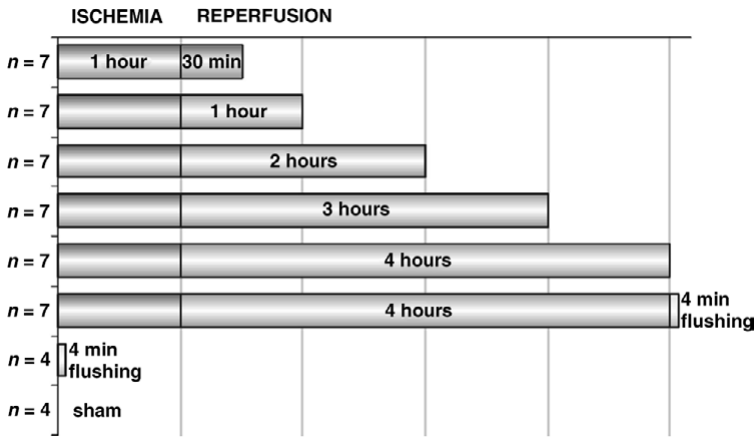
Experimental setting.

Fifty rats were randomized into eight groups. Five groups underwent 1 hour of warm lung ischemia followed by 30 min, 1, 2, 3 and 4 hours of reperfusion, respectively (*n *= 7 in each group). One sham group underwent the identical surgical procedure without ischemia–reperfusion (*n *= 4). To find out whether adhesion of the inflammatory cells had occurred, the lungs in one extra group were flushed with 6% buffered hetastarch after 1 hour of ischemia and 4 hours of isolated lung perfusion (*n *= 7) [14]. This group was compared with a sham group, which was also flushed (*n *= 4) (Fig. 1).

### Induction of ischemia–reperfusion

Anesthesia was induced by 4% isoflurane in a mixture of oxygen (O_2_) and nitrous oxide (N_2_O) in a ratio of 1:3 for 4 min. Intubation was performed with a 16-gauge Insyte-W catheter using translaryngeal illumination in accordance with the technique described by Hendriks [14]. After the rats had been connected to the ventilator, the N_2_O : O_2 _ratio was set to 1:1 and the concentration of isoflurane was titrated to 0.5–1.5% according to muscle relaxation, heart rate and pupil size. To prevent thrombosis in lung vasculature during ischemia, 100 IU/kg heparin was infused into the left femoral vein 5 min before the left lung hilum was clamped. After left posterolateral thoracotomy through the fourth intercostal space, a rib retractor was placed to luxate the left lung anteriorly.

Ischemia was induced by clamping the left lung hilum with two occluding curved microvascular clamps (Kleinert-Kurz WK65145) without further dissection. One clamp was placed in a cranial–caudal direction and the other clamp was placed laterally in the opposite direction. In a separate experiment, four rats received intravenous and bronchial injection of methylene blue solution to test the vascular and bronchial occlusion obtained by the microvascular clamps. Complete vascular and bronchial occlusion was achieved. To simulate physiological circumstances, the thoracotomy incision was closed in layers after the introduction of a 16-gauge catheter connected to a 50 ml syringe into the left chest cavity.

When animals recovered, the chest tube and endotracheal tubes were removed. Ten minutes before reperfusion, anesthesia was induced and rats underwent a left thoracotomy with the use of the same incision as described above. Reperfusion occurred on removal of the clamps. The left thoracotomy was closed as described above. Ten minutes before the end of reperfusion time anesthesia was induced and the rat underwent a left thoracotomy for the third time followed by left pneumonectomy. Ten seconds before the rat was killed, maximal inflation of the left lung was achieved by occlusion of the expiratory ventilation cannula for 3 s to prevent inter-animal variation of inflation of the left lung. After reperfusion all rats underwent intramuscular injection of tramadol for pain control.

To prevent cooling, rats were placed on a warm-water pad during the operation and under a heating light during both ischemia and reperfusion. Rectal temperature was measured before clamping of the left lung hilum and before killing and was held constantly between 36.8 and 37.4°C.

Rats in the sham group underwent an identical surgical procedure except for clamping the left lung hilum. Rats in this group were killed 1 h after anterior luxation of the left lung.

### Flush procedure

To study cellular adhesion to the endothelium, lungs of one more group were flushed after 4 hours of isolated lung perfusion with buffered starch. This procedure has been extensively described previously [15,16]. In brief, after ischemia–reperfusion, the pulmonary artery and vein were clamped with curved microvascular clamps. A 16-gauge angiocatheter was placed through the chest wall. A PE-10 perfusion catheter (Clay Adams, Parsippany, NJ, USA) was introduced into the chest through the angiocatheter and secured by a 4/0 silk suture after insertion into the pulmonary artery. Perfusate (6% buffered starch) was delivered through this catheter for 4 min at 0.5 ml/min. In addition, a pulmonary venotomy was performed to discard the venous effluent.

### Killing and tissue storage

At killing, the left lung was taken out of the rat and cut caudal–cranially into four pieces. The lateral sample was fixed in metacarn for 4 hours at room temperature (23°C) and stored in 70% ethanol at 4°C. Directly after killing, the weight of the medial sample was measured and the sample was put into an oven at 65°C for 5 days to assess the wet : dry ratio as a parameter for lung oedema. The middle samples were fixed in chloroform calcium for 90 min at room temperature and then stored in buffer (10 ml of distilled water, 1 g of CaCl_2_, 0.121 M cacodylate) at 4°C until further processing. Killing was performed by a cut down of the superior caval vein.

### Sample processing

Tissue samples for light-microscopic investigations were dehydrated with propan-2-ol, cleared with toluene and embedded in paraffin wax. Sections 4 μm thick were stained with hematoxylin/eosin stain (H&E) for neutrophil count. Immunohistochemistry was applied for macrophage and T cell visualization. After deparaffination, endogenous peroxidase was blocked by incubation in 0.9% H_2_O_2 _for 15 min. The sections were incubated overnight with CD-3-specific antibody 1F4 (Pharmingen, Becton Dickinson, Erembodegem, Belgium) or with antibody ED-1 (Serotec, Diagnostic Products Cooperation, Humbeek, Belgium) directed against lysosomal membrane glycoprotein on macrophages. Incubation for 30 min with secondary biotinylated horse anti-mouse antibody (Vector, Burlingame, CA, USA) was followed by incubation for 1 hour with peroxidase-labeled avidin–biotin complex (Vector). The slides were developed in 3,3-diaminobenzidine with 0.03% H_2_O_2 _or 3-amino-9-ethylcarbazole (AEC) with 0.006% H_2_O_2 _for 30 min. Finally, counterstaining was performed in methyl green and Haemaluin Carazzi to reveal T cells and macrophages, respectively.

### Light-microscopy investigation

**Figure 2:**
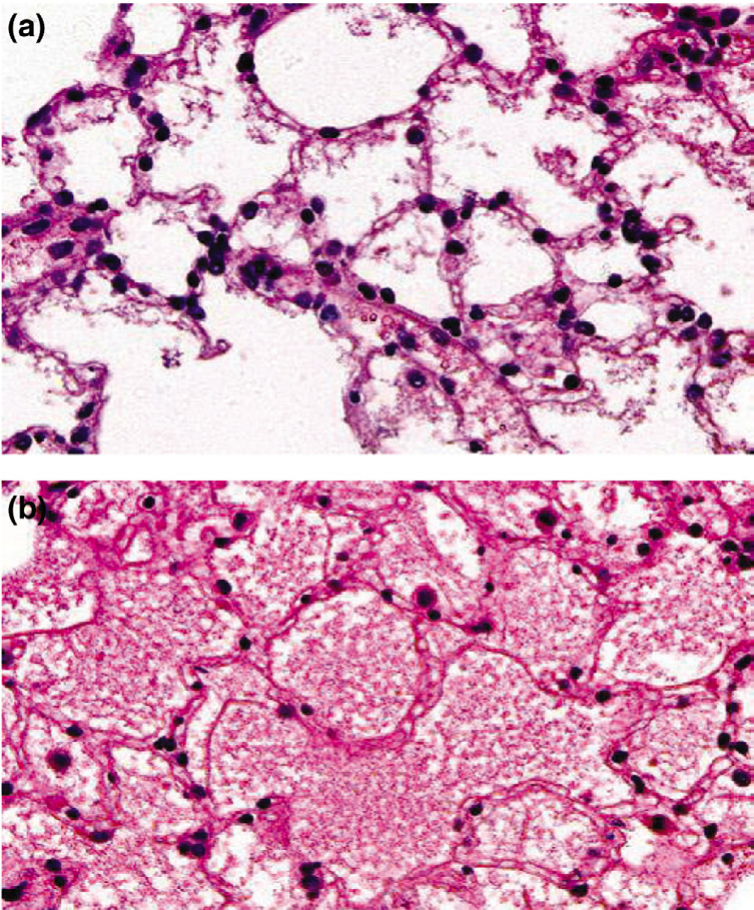
Mild **(a) **and severe **(b) **alveolar oedema after 1 hour of warm pulmonary ischemia followed by 4 hours of reperfusion; hematoxylin/eosin stain.

All slides were evaluated in random order. The first field was chosen at random and the next fields in accordance with a standard pattern. Neutrophils were counted in 20 fields per slide (0.95 mm^2 ^per slide, magnification ×500). Macrophages were counted in 30 fields per slide (5.65 mm^2 ^per slide, magnification ×250). T cells were counted in 20 fields per slide (1.54 mm^2 ^per slide, magnification ×400). Apoptosis was determined by terminal deoxynucleotidyl transferase-mediated (TdT) dUTP nick end labelling (TUNEL) staining. Deparaffinization was performed as described above. After decalcification with 3% citrate dissolved for 1 hour at 37°C, sections were incubated with TdT (Roche, Brussels, Belgium) in combination with fluorescein isothiocyanate-labelled dUTP nucleotides (AP Biotech, Roosendaal, The Netherlands) for 1 hour at room temperature. Furthermore, incubation with anti-fluorescein isothiocyanate (Dako, Glostrup, Denmark) peroxidase was performed followed by subsequent washes and the specimens were stained in AEC and counterstained with Haemaluin Carazzi. Only cells with TUNEL-positive nuclear and no cytoplasmic staining were considered to be apoptotic. Cells containing positive cytoplasmic staining were not counted. TUNEL-positive fragments closely ordered in a group were defined as apoptotic bodies. Apoptotic bodies and cells were both counted in 20 fields per slide (0.23 mm^2 ^per slide, magnification ×800). The occurrence of necrosis was investigated in H&E by a pathologist (EvM) who did not have any knowledge of details of the study. Oedema was twice assessed blindly at H&E and was graded, ranging from mild, moderate to severe. Mild oedema was defined as no to slight exudation within the alveolar space (Fig. 2a). Severe oedema was defined as easily recognizable full exudation in the alveolar space (Fig. 2b); moderate oedema was defined as being between mild and severe.

### Statistics

All statistics were performed with SPSS 9.0 for Windows. Cellular infiltrates and apoptosis were evaluated statistically with the Kolmogorov–Smirnov test to confirm normal distribution. Analysis of variance and Student's *t*-test were applied to compare data obtained from the different reperfusion periods with the sham groups. Graded oedema frequencies were analyzed with the χ^2 ^test by comparison of the reperfusion groups with the sham groups. Statistical significance was accepted at *P *< 0.05.

## Results

### Cellular infiltrations

#### Neutrophils (H&E, magnification ×500)

**Figure 3:**
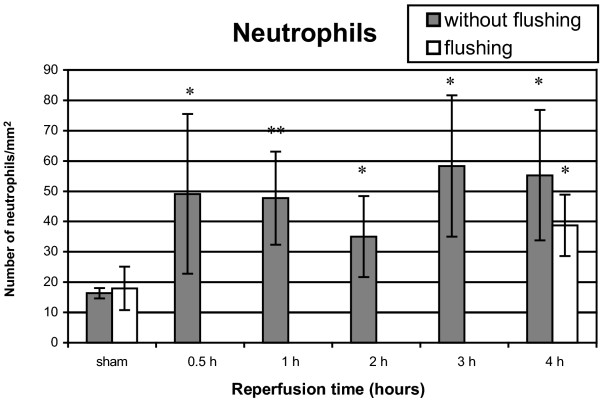
Neutrophil infiltration after 1 hour of warm pulmonary ischemia followed by 30 min to 4 hours of reperfusion. Results are expressed as neutrophils/mm^2 ^and are means ± SD. *P < 0.01; **P < 0.001.

A significant increase in neutrophils was observed after 30 min to 4 hours reperfusion compared with the sham group (*P *< 0.01) (Fig. 3). After 4 hours of reperfusion followed by flushing, significantly more neutrophils were counted than in the flushed sham group (*P *= 0.003), whereas no significant difference was observed compared with 4 hours of reperfusion without flushing (*P *= 0.10).

#### Macrophages (ED-1, magnification ×250)

**Figure 4:**
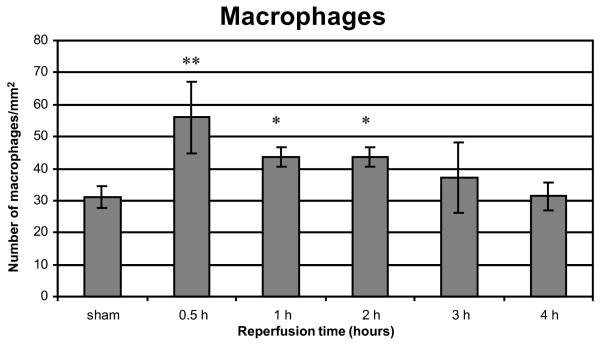
Macrophage infiltration after 1 hour of warm pulmonary ischemia followed by 30 min to 4 hours of reperfusion. Results are expressed as macrophages/mm^2 ^and are means ± SD. *P < 0.01; **P < 0.001.

Significantly more macrophages were counted after 30 min of reperfusion (*P *= 0.0002), 1 hour (*P *= 0.004) and 2 hours (*P *= 0.007) of reperfusion compared with the sham group (Fig. 4). A significant decrease was observed after 1 hour of reperfusion compared with 30 min of reperfusion (*P *= 0.01). After 3 hours (*P *= 0.06) and 4 hours (*P *= 0.61) of reperfusion no significant increase in macrophages was observed compared with the sham group.

#### T cells (1F4, magnification ×400)

**Figure 5:**
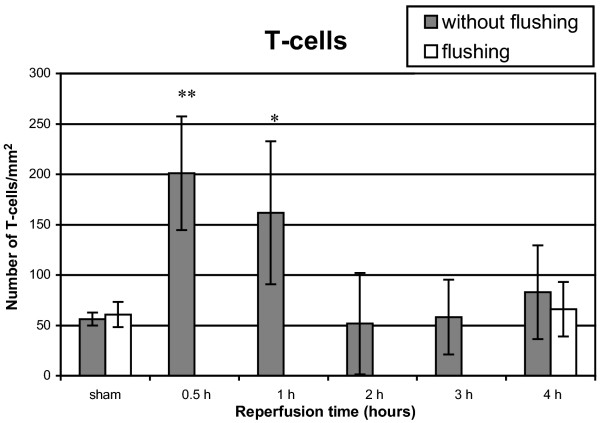
T cell infiltration after 1 hour of warm pulmonary ischemia followed by 30 min to 4 hours of reperfusion. Results are expressed as T cells/mm^2 ^and are means ± SD. *P < 0.01; **P < 0.001.

A fourfold increase of T cells was observed after 30 min of reperfusion (*P *= 0.0002) compared with the sham group (Fig. 5). This increase was also significant after 1 hour of reperfusion (*P *= 0.004). From 2 hours to 4 hours no significant increase was observed.

### Injury evaluation

#### Apoptosis (TUNEL, magnification ×800) and necrosis (H&E)

**Figure 6:**
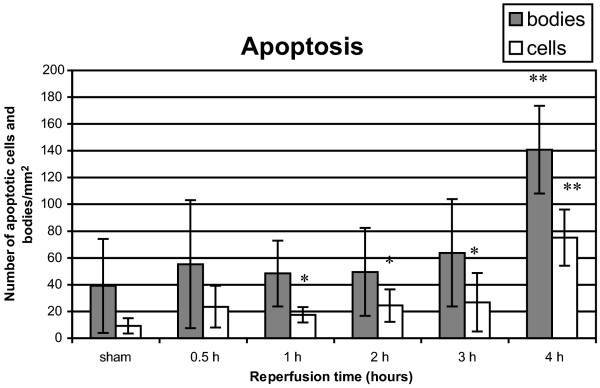
Apoptotic cells and bodies after 1 hour of warm pulmonary ischemia followed by 30 min to 4 hours of reperfusion. Results are expressed as apoptotic cells and bodies/mm^2 ^and are means ± SD. *P < 0.05; **P < 0.001.

Significantly more apoptotic cells were seen after 1 hour (*P *= 0.03), 2 hours (*P *= 0.01), 3 hours (*P *= 0.04) and 4 hours (*P *= 0.00004) of reperfusion (Fig. 6). The number of apoptotic bodies was significantly higher after 4 hours of reperfusion (*P *= 0.0006).

Necrosis was not observed in any group.

#### Oedema (H&E)

**Figure 7:**
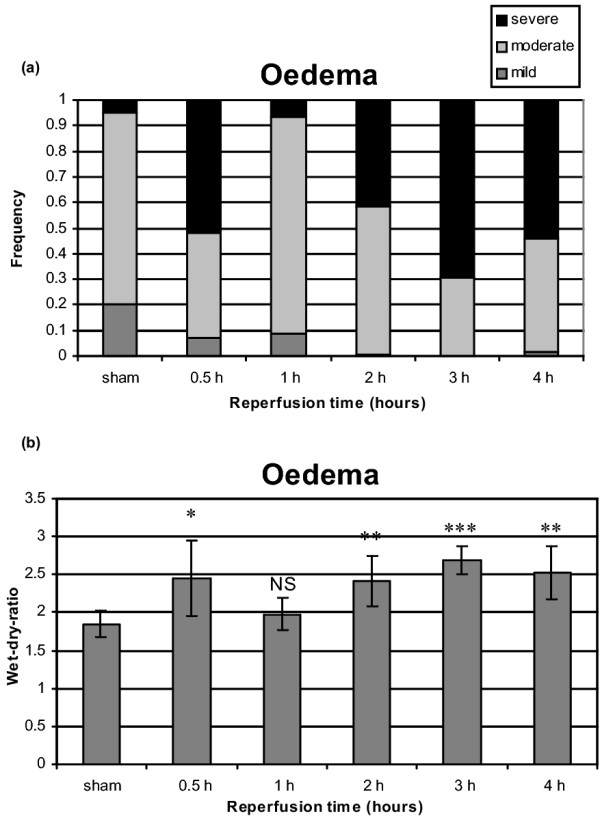
Alveolar oedema after 1 hour of warm pulmonary ischemia followed by 30 min to 4 hours of reperfusion.**(a) **Histological assessment of alveolar oedema in H&E. **(b) **Wet : dry ratio. *P < 0.05; **P < 0.01; ***P < 0.001.

Histological examination showed significantly more alveolar oedema after 30 min, 2, 3 and 4 hours of reperfusion (*P *< 0.0001) compared with the sham group (Fig. 7a). However, after 1 hour of reperfusion, oedema was not significantly increased compared with the sham group. The wet : dry ratio was significantly increased in all groups (30 min, *P *< 0.05; 2 hours, *P *< 0.01; 3 hours, *P *< 0.001; 4 hours, *P *< 0.01) except for 1 hour of reperfusion (Fig. 7b).

## Discussion

In this study a significant increase in neutrophils was observed after 1 hour of warm ischemia followed by 30 min to 4 hours of reperfusion. A first peak was shown after 30 min of reperfusion and a second peak after 3 hours of reperfusion. Furthermore, after 4 hours of reperfusion, significantly more neutrophils were observed after pulmonary artery flushing than in the flushed sham group. This resulted in flushing of cells that did not adhere to the endothelium. These results suggest activation and adhesion of neutrophils to the endothelium. Our observations are partly in contrast with results of Eppinger and colleagues, who showed a bimodal pattern of lung injury after 90 min of warm ischemia, with a first peak after 30 min of reperfusion and a second peak after 4 hours of reperfusion [17]. In their report, myeloperoxidase activity, representing neutrophil sequestration, diminished during the reperfusion time course. Neutrophil depletion did not have a protective effect on microvascular permeability after 30 min of reperfusion but the authors did show a protective effect after 4 hours, suggesting an early neutrophil-independent phase and a late neutrophil-dependent phase [17]. The observation of late neutrophil-dependent lung injury is indirectly related to our observation that significantly more neutrophils were counted after flushing of non-adhesive cells, suggesting activation of these cells.

The role of macrophages has been investigated only in transplantation models [3,10,11]. Our data show significantly more macrophages after 30 min to 2 hours of reperfusion, which is in accordance with data from Eppinger. Using the permeability index Eppinger showed an attenuation of reperfusion injury using antibodies against monocyte chemoattractant protein-1, TNF-α and interferon-γ, suggesting that reduced early reperfusion injury is probably due to suppression of macrophage function [10]. A recent report by Maxey and colleagues confirmed the central role of macrophages in early reperfusion injury. They demonstrated significantly less lung injury in TNF-α-deficient mice after 1 hour of ischemia and 1 hour of reperfusion, suggesting that TNF-α is a key initiating factor in acute lung injury [18].

Fiser has made a distinction between the role of donor macrophages on the one hand and the role of recipient macrophages on the other. Activation of donor macrophages could be the initial consequence of ischemia and early reperfusion. In reaction to activation, donor macrophages deliver cytokines, chemotactic agents and proteolytic enzymes responsible for early reperfusion injury [3,11]. Subsequently, early lung injury activates the inflammatory mechanisms of the recipient [10].

Beside augmentation of neutrophils and macrophages, our study also showed a fourfold (*P *= 0.0002) increase in T cells after 30 min to 1 hour of reperfusion, followed by a rapid attenuation. Because of the short duration of reperfusion it is unlikely that local proliferation of lymphocytes occurred, suggesting that chemotaxis is responsible for these observations. However, it is not clear that activation of these cells happened because of the rapid attenuation after 2 hours of reperfusion. This finding implies that the early augmentation of lymphocytes is just a non-specific inflammatory reaction on early reperfusion injury.

The role of T cells was investigated recently in a model of mouse lung perfusion with fresh blood [19]. The interaction between allogenic blood lymphocytes and vascular endothelial cells is correlated with high expression of mRNA of both adhesion molecules and TNF-α in the perfused lung, suggesting that antigen-dependent activation of lymphocytes had occurred [19].

To our knowledge the present study is the first to show apoptosis in the absence of necrosis in lung tissue after warm ischemia–reperfusion. An explanation for the absence of necrosis after 4 hours of reperfusion might derive from the length of reperfusion. Experiments with longer reperfusion periods will be necessary to confirm this hypothesis.

The number of apoptotic bodies is significantly increased after 1–4 hours of reperfusion, whereas the number of apoptotic cells is significantly increased after 4 hours of reperfusion. The tendency of apoptosis to increase is in accordance with observations of Fischer and colleagues in a human transplantation study with 1–5 hours of cold ischemia that showed significant increases in the number of apoptotic cells after reperfusion, in a time-dependent manner [13]. In particular, alveolar type II pneumocytes seemed to be apoptotic [13]. Stammberger and colleagues reported a peak of apoptotic cells after 18 hours of cold ischemia and 2 hours of reperfusion followed by a quick decrease in apoptotic cells as a function of reperfusion time [20]. The rapid attenuation of apoptotic cells is probably due to the occurrence of apoptosis after 6–12 hours of preservation and especially necrosis after 18–24 hours of preservation as described by Fischer and colleagues [21]. Furthermore, an inverse correlation of the occurrence of necrosis with oxygenation was shown, implying the necessity of preventing necrosis [21].

This study showed an identical pattern of alveolar oedema in a function of time by using a histological examination (H&E) and assessment by wet : dry ratio. An important increase of alveolar oedema was observed after 30 min, 2, 3 and 4 hours of reperfusion. We do not have an explanation for the absence of significant oedema after 1 hour of reperfusion. However, a bimodal pattern of lung injury reported by Eppinger and colleagues [17] is confirmed by our results. Using the vascular permeability of ^125^I-labeled bovine serum albumin, Eppinger and colleagues showed an increased presence of serum albumin in bronchoalveolar lavage after 90 min of warm ischemia followed by a first peak after 30 min of reperfusion and a second peak after 4 hours of reperfusion, indicative of damage to the normal vascular/airway barrier [17].

Pulmonary ischemia results histologically in alveolar oedema due to changing permeability at the blood/air barrier after only 30 min of reperfusion. Apoptotic cells appear after 4 hours of reperfusion in a warm model of ischemia–reperfusion and after 6–9 hours of reperfusion in a transplantation model, whereas necrosis is observed after 18–24 hours of reperfusion related to an inverse correlation with oxygenation [21]. It may be noticed that these observations are related to a clinical feature known as ARDS. Clinical ARDS is characterized by acute hypoxemic respiratory failure due to non-cardiogenic pulmonary oedema caused by increased permeability of the alveolar capillary barrier, resulting in mortality ranging from 35% to 44% [22]. On the basis of the results of this study, research has to be focused on how cellular infiltrates are involved in the occurrence of ARDS and in what manner intervention might diminish the damaging effect of pulmonary ischemia–reperfusion.

## Conclusion

This study has shown a significant increase in neutrophils after 30 min to 4 hours of reperfusion as well as after reperfusion followed by flushing. Macrophages doubled in number in lung tissue after ischemia–reperfusion. A fourfold increase in T cells in lung tissue after 1 hour of warm ischemia and 30 min of reperfusion was observed. Furthermore, apoptosis in the total absence of necrosis was shown together with important alveolar oedema.

## Key messages

• 	Significant early increase of T-cells macrophages and neutrophils after 1 hour of ischemia and 4 hours of reperfusion

• 	Significant late increase of neutrophils after 1 hour of ischemia and 4 hours of reperfusion.

• 	Significant apoptosis and lung oedema in the absence of necrosis after 1 hour of ischemia and 4 hours of reperfusion.

## Abbreviations

AEC = 3-amino-9-ethylcarbazole; ARDS = acute respiratory distress syndrome; H&E = hematoxylin/eosin stain; TNF = tumor necrosis factor; TUNEL = TdT-mediated dUTP nick end labelling.

## Competing interests

The author(s) declare that they have no competing interests.

## Authors' contributions

BVP and JH performed all surgical procedures under the supervision of PVS. BVP and VP performed histological analyses of the lung specimens under the supervision of EvM and MDB. VP also performed statistical analyses. BVP drafted the manuscript and was advised by JK. All authors read and approved the final manuscript.
